# Structural and Functional Alterations of Substantia Nigra and Associations With Anxiety and Depressive Symptoms Following Traumatic Brain Injury

**DOI:** 10.3389/fneur.2022.719778

**Published:** 2022-04-05

**Authors:** Liang Gao, Qiang Xue, Shun Gong, Gaoyi Li, Wusong Tong, Mingxia Fan, Xianzhen Chen, Jia Yin, Yu Song, Songyu Chen, Jingrong Huang, Chengbin Wang, Yan Dong

**Affiliations:** ^1^Department of Neurosurgery, Shanghai Tenth People's Hospital, Tongji University School of Medicine, Shanghai, China; ^2^Department of Neurosurgery, Eastern Hepatobiliary Surgery Hospital, Naval Medical University, Shanghai, China; ^3^Department of Neurosurgery, General Hospital of Northern Theater Command, Shenyang, China; ^4^Department of Neurosurgery, People's Hospital of Putuo District, Tongji University School of Medicine, Shanghai, China; ^5^Department of Neurosurgery, Shanghai Pudong New Area People's Hospital, Shanghai, China; ^6^Shanghai Key Laboratory of Magnetic Resonance, East China Normal University, Shanghai, China; ^7^Psychology Honors Program, University of California, San Diego, San Diego, CA, United States; ^8^Shanghai Tenth People's Hospital Clinical Medicine Scientific and Technical Innovation Park, Shanghai, China

**Keywords:** traumatic brain injury, functional connectivity, dopamine, anxiety, depression, substantia nigra

## Abstract

**Backgrounds:**

Although there are a certain number of studies dedicated to the disturbances of the dopaminergic system induced by traumatic brain injury (TBI), the associations of abnormal dopaminergic systems with post-traumatic anxiety and depressive disorders and their underlying mechanisms have not been clarified yet. In the midbrain, dopaminergic neurons are mainly situated in the substantia nigra (SN) and the ventral tegmental area (VTA). Thus, we selected SN and VTA as regions of interest and performed a seed-based global correlation to evaluate the altered functional connectivity throughout the dopaminergic system post-TBI.

**Methods:**

Thirty-three individuals with TBI and 21 healthy controls were recruited in the study. Anxiety and depressive symptoms were examined by the Hospital Anxiety and Depression Scale. All MRI data were collected using a Siemens Prisma 3.0 Tesla MRI system. The volume of SN and the global functional connectivity of the SN and VTA were analyzed.

**Results:**

In the present study, patients with TBI reported more anxiety and depressive symptoms. More importantly, some structural and functional alterations, such as smaller SN and reduced functional connectivity in the left SN, were seen in individuals with TBI. Patients with TBI had smaller substantia nigra on both right and left sides, and the left substantia nigra was relatively small in contrast with the right one. Among these findings, functional connectivity between left SN and left angular gyrus was positively associated with post-traumatic anxiety symptoms and negatively associated with depressive symptoms.

**Conclusions:**

The TBI causes leftward lateralization of structural and functional alterations in the substantia nigra. An impaired mesocortical functional connectivity might be implicated in post-traumatic anxiety and depression.

## Introduction

Anxiety and depressive disorders are common psychiatric sequelae, following traumatic brain injury (TBI), and have major detrimental impacts on social functions and cognitive outcomes (1). While previous studies have already demonstrated the roles of serotonergic and GABAergic neurotransmitter systems in anxiety and depression, multiple lines of evidence also suggest the involvement of the dopaminergic circuit in the pathology of anxiety and depressive disorders (2, 3). Depression and anxiety are frequent non-motor features in Parkinson's disease (PD) and can be present during the prodromal phase of PD (4, 5). Dopamine transporter (DAT) imaging establishes the associations of dopamine deficits in the mesolimbic pathway with anxiety and depression in PD (6, 7). Thobios et al. reported that anxiety and depression, which occurred as a delayed dopamine withdrawal syndrome following subthalamic nucleus stimulation, are related to a varying extent of mesocortical and mesolimbic dopaminergic denervation (8). Later on, more neuroimaging studies further support the idea that extensive structural and functional disruptions in the dopamine mesolimbic and mesocortical pathway might be highly implicated in the pathology of anxiety and depression (9–11).

The TBI can damage the dopaminergic systems. Early clinical observations show that boxers, football players, and patients with TBI are at an increased risk of developing Parkinson-like symptoms, which shed light on the association of TBI with disrupted dopaminergic neurons (12, 13). Subsequent studies suggest that both single and repetitive TBI can contribute to biochemical dysregulation of the dopaminergic system and dopaminergic neuronal loss in the midbrain (14–16). Recently, neuroimaging data has demonstrated reduced DAT levels and dopamine D2/D3 receptor availability in the striatum, along with damage within the substantia nigra (SN) and nigrostriatal projections (17–19). In PD, the neuronal loss in the SN can be asymmetric. Neuroimaging evidence demonstrates that the left nigrostriatal dysfunction is predominant in right-handed patients with PD (20, 21). However, it is unknown whether it is the case for TBI. Although there is a certain number of studies dedicated to the disturbances of the dopaminergic system induced by TBI, the associations of the abnormal dopaminergic system with post-traumatic anxiety and depressive disorders, and their underlying mechanisms have not been clarified yet.

In the midbrain, dopaminergic neurons are mainly situated in the SN and the ventral tegmental area (VTA), which have a variety of projections to striatal, subcortical, and cortical areas *via* nigrostriatal, mesolimbic, and mesocortical pathways. Recent studies have mapped the connectivity patterns of DA midbrain nuclei using resting-state functional MRI (rs-fMRI) connectivity and evaluated the maturation of functional connectivity of VTA and SN from childhood to adulthood (22, 23). In the present study, we selected SN and VTA as regions of interest (ROI) and performed a seed-based global correlation to evaluate the altered functional connectivity throughout the dopaminergic system post-TBI. Moreover, the bilateral SN volumes were measured. We aimed to explore the structural and functional alterations following TBI and their correlations with post-traumatic anxiety and depressive symptoms.

## Materials and Methods

### Participants

This was a cross-sectional observational study conducted from October 2018 to June 2019. The patients were eligible for recruitment if they presented with the following characteristics: older than 18 years; with first-ever TBI and positive finding on cranial CT scans on admission; without neurological or psychiatric disorders prior to TBI. The initial evaluation of the severity of TBI took place within the first 24 h after an injury during hospitalization based on Glasgow Coma Scale (GCS), which classifies TBI into three categories: mild (GCS, 13–15), moderate (GCS, 9–12), and severe (GCS, 3–8). Healthy participants without a history of TBI and neurological and psychiatric disorders were matched for age, gender, and education. All the participants were right-handed. Thirty-three patients with TBI and 21 healthy controls were included in the study. The ethics committee of the Pudong New Area People's Hospital approved the study. Informed written consent for study participation was obtained from all the patients and healthy controls.

### Neuropsychological Assessment

The Hospital Anxiety and Depression Scale (HADS), which is a brief self-assessment questionnaire measuring the severity of the emotional disorder and has been validated in populations with TBI, was employed to evaluate the anxiety and depressive symptoms. The anxiety and depression subscales have seven items, respectively. A Wechsler Memory Scale-Chinese Revision (WMS-CR) picture, recognition, associative learning, comprehension memory, and digit span were administered to evaluate multiple categories of memory capacity. The sum of five subscales was calculated to reflect the general memory function. An overall global outcome was evaluated by a standard interview using the Glasgow Outcome Scale-extended (GOS-E).

### Image Acquisition

All MRI data were collected using a Siemens Prisma 3.0 Tesla MRI system (Prisma, Siemens, Erlangen, Germany), equipped with a 20-channel head coil. The participants lay in a supine position in the MRI scanner with cushions to restrict the mobility of their heads, which minimizes the head motion. During rs-fMRI scanning, the participants were guided to stay awake with their eyes closed without thinking about anything in particular. The Rs-fMRI data were obtained using an echo-planar imaging sequence with 240 contiguous EPI functional volumes, with 33 axial slices, slice thickness = 3.5 mm, gap = 0.7 mm, TR/TE = 2,000/30 ms, flip angle = 90°, field of view = 224 mm × 224 mm, matrix size = 64 × 64; voxel size = 3.5 mm^3^ × 3.5 mm^3^ × 3.5 mm^3^. The total rs-fMRI scan length was 8 min 6 s with the first 6 s was consumed on the dummy scan. The structural images were acquired using a high-resolution T1-weighted magnetization prepared rapid acquisition gradient-echo (MPRAGE) sequence with 192 sagittal slices, TR/TE = 2,530/2.98 ms, flip angle = 7°, FOV = 256 mm × 256 mm, matrix size = 256 × 256, voxel size = 1 mm^3^ × 1 mm^3^ × 1 mm^3^, which facilitated the localization and co-registration of functional data. Also, transverse turbo-spin-echo T2-weighted images for lesion localization were obtained with 30 axial slices, slice thickness = 5 mm, TR/TE = 6,000/95 ms, flip angle = 120°, FOV = 220 mm × 220 mm, matrix size = 320 × 320, voxel size = 0.34 mm^3^ × 0.34 mm^3^ × 5 mm^3^.

### Resting-State fMRI (rs-fMRI) Data Processing

Functional images were processed using the Data Processing Assistant for Resting-State fMRI (DPARSF) toolbox (http://rfmri.org/DPARSF), which provides a standard data preprocessing pipeline for resting-state and task-based fMRI, including Digital Imaging and Communications in Medicine (DICOM) to Neuroimaging Informatics Technology Initiative (NIfTI), slice timing, realignment, nuisance regression, spatial normalization, and smoothing (24). Transformations from individual native space to Montreal Neurological Institute (MNI) space were computed with the Diffeomorphic Anatomical Registration Through Exponentiated Lie algebra (DARTEL) tool.

Nuisance regression was implemented as previously described (25). The head motion effects were regressed out using the Friston 24-parameter motion correction. Mean framewise displacement (FD, derived from Jenkinson's relative root mean square algorithm) was employed to address the residual effects of motion as a covariate in group analyses. In validation analysis, scrubbing (removing time points with FD >0.2 mm) was also utilized to verify results using an aggressive head motion control strategy. To reduce respiratory and cardiac confounds, other sources of spurious variance [White Matter (WM) and Cerebrospinal Fluid (CSF) signals] were also removed from the data through linear regression. Furthermore, we included the linear trend as a regressor to account for drifts in the blood-oxygen-level-dependent (BOLD) signal. Spatial smoothing with a Gaussian kernel of 4-mm full-width half maximum (FWHM) was applied to the functional images. Temporal bandpass filtering (0.01–0.1 Hz) was performed to reduce the effect of low-frequency drift and high-frequency noise.

Based on Volkow ND's data, three ROIs were selected to represent the location of VTA [MNI coordinates: (0, −15, −12) mm] and SN [MNI coordinates: (±12, −12, −12) mm] (22). All ROIs were surrounded by a 3-mm radius sphere. A seed-based correlation approach was performed to establish the networks that are functionally connected to VTA and SN. Pearson correlations were used to compute the strength of the functional connectivity between the BOLD signals at the seed locations and those in other brain voxels, and the Fisher transform was used to convert the correlation coefficients into normally distributed coefficients.

### Substantia Nigra (SN) Volume Measurement

The substantia nigra was segmented using T2-weighted images in 3D Slicer. Compared to T1-weighted sequences, the iron load is an advantage in T2-weighted sequences, which provides enhanced contrast and facilitates visualization of SN contours and shapes (26). We manually delineated the outline of bilateral SNs at the midbrain level according to the dark signals of ventral red nucleus in anatomical T2 space using the 3D Slicer Editor module and reconstructed the right SN and left SN models using the Model Maker module. The segmentation results were checked by multiple expert readers (SG, YD, and QX) using T1/T2-weighted images and fluid-attenuated inversion recovery images.

The voxel volume of the SN was calculated as follows: We transformed the SN into a label map (a segmented image volume; in this case, a binary mask) in T2 space in 3D Slicer. This module sets a specified label value in the label map at every vertex of bilateral SNs. Then, we applied the Label Statistics module to calculate the label volume. The volume of the SN was defined as the volume of the voxels occupied by the SNs in each subject.

### Total Intracranial Volume Measurement

T1-weighted images were processed by using Statistical Parametric Mapping software (http://www.fil.ion.ucl.ac.uk/spm/) and the Computational Anatomy Toolbox (http://dbm.neuro.unijena.de/cat12/). Briefly, the T1-weighted images were corrected for the bias field inhomogeneities, and then, segmented into different components, including gray matter, white matter, and cerebral spinal fluids, followed by spatially normalization into MNI space by the DARTEL algorithm. Total intracranial volume (TIV) was calculated as the sum of gray matter, white matter, and cerebrospinal fluid volumes.

### Statistical Analyses

Statistical analyses were conducted using statistical product and service solutions (SPSS), Version 22.0 (IBM, Armonk, New York), and DPARSF. Continuous variables were described using means and SDs, and categorical variables were summarized using frequencies. The normality distribution of continuous data was checked with a one-sample Kolmogorov-Smirnov test. The independent *t*-test and Mann–Whitney U-test were applied to perform the group comparisons for normally distributed and non-normally distributed data, respectively. The between-group gender was computed using a Chi-square test.

Group comparisons for functional connectivity were analyzed using DPARSF with a two-sample *t*-test, controlling for age, gender, education, mean FD, and gray matter volume. Correction for multiple comparisons was performed by Gaussian random field correction (GRF) using a voxel-level threshold of *p* < 0.001 and a cluster-level threshold of *p* < 0.05. A set of analysis of covariance (ANCOVA) models was applied to compare group differences on SN volume, adjusting gender, age, education, and TIV. A paired *t*-test was applied to examine the difference between right and left SN volumes. Relations of functional connectivity with HADS anxiety and depression were examined in multiple linear regressions. Results were considered significant if *p*-values were <0.05.

## Results

Due to head motion outliers (FD >0.2 mm) on the rs-fMRI scanning, three individuals with TBI and one healthy control were excluded. Finally, 30 individuals with TBI and 20 healthy controls were included in the final analyses. The mean FD for patients with TBI (mean = 0.11, SD = 0.04) was not different from that for healthy controls (mean = 0.11, SD = 0.05) (*p* = 0.874).

### Demographic and Clinical Characteristics

**Figure 1:**
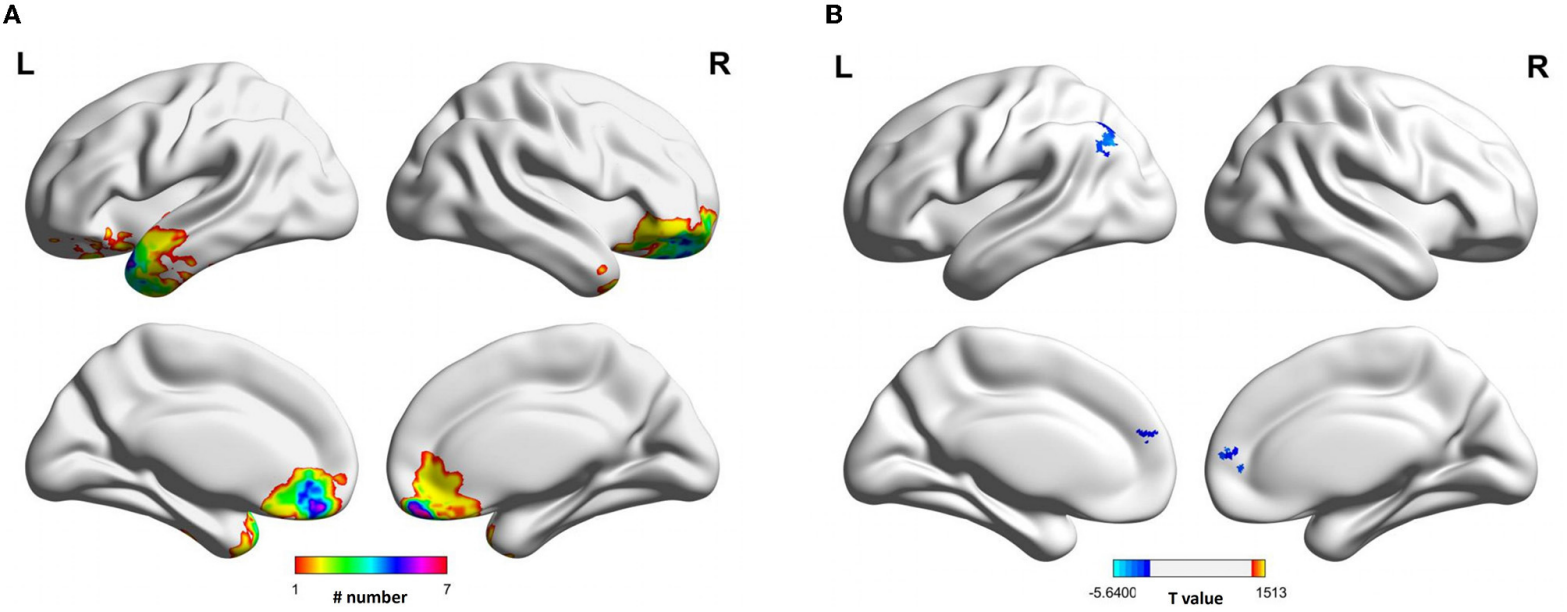
Lesion map and functional connectivity of left substantia nigra following traumatic brain injury. **(A)** The focal lesions visible on MRI scannings were mainly overlapped in the anterior frontal and temporal lobes. **(B)** The functional connectivity between left substantia nigra and bilateral superior medial frontal gyrus, and left angular gyrus was decreased in TBI group.

Table 1 summarizes the demographic and clinical characteristics of all the participants. The mean age was similar between the groups (*p* = 0.416). No Differences in gender and education between groups were observed. All the patients showed CT-positive findings on admission, and 23 patients showed one or more intracerebral lesions on follow-up MRI, with high overlap in the prefrontal and anterior temporal cortex (Figure 1A). More than half of the patients had a GOS-E score of 6 or lower at follow-up. Compared to healthy controls, the patients with TBI reported more anxiety and depressive symptoms and performed more poorly on memory tests.

**Table 1:** Demographics and clinical characteristics in patients with traumatic brain injury (TBI) and healthy controls.

	**TBI (*n* = 30)**	**Healthy control (*n* = 20)**	***P*-value**
**Demographic information**			
Age, mean (SD), y	35.30 (10.33)	32.95 (9.25)	0.416
Male, No. (%)	19 (63.33)	14 (70)	0.763
Educational lever, mean (SD), y	9.20 (4.29)	9.15 (3.86)	0.967
**Injury-related information**			
GCS, No. (%)		NA	–
13–15	20 (66.67)		
9–12	5 (16.67)		
3–8	5 (16.67)		
Intracerebral lesion on MRI scan, No. (%)		NA	
Yes	23(76.67)		
No	7(23.33)		
**Neuropsychological measurements**			
GOS-E, mean (SD)	6.07 (1.36)	NA	
HADS anxiety, mean (SD)	8.23 (4.84)	4.40 (2.54)	0.001
HADS depression, mean (SD)	7.63 (5.83)	2.85 (2.18)	0.000
Memory, mean (SD)	39.72 (13.92)	51.95 (7.44)	0.000

*TBI, traumatic brain injury; GCS, Glasgow coma scale; GOS-E, Glasgow outcome scale-extended; NA, not applicable; HADS, hospital anxiety and depression scale*.

### Functional Connectivity

The MNI spheres were chosen as ROIs to localize the bilateral SN and VTA. The seed-based global functional connectivity analyses showed that the participants with TBI presented reduced functional connectivity in the left SN compared to healthy controls (Table 2). The weakened functional connectivity between the left SN and bilateral superior medial frontal gyrus, extending into the right anterior cingulate cortex, was seen. Additionally, the left SN showed reduced functional connectivity to the left angular gyrus, extending into the left inferior parietal lobe (Figure 1B). Group comparisons on functional connectivity of right SN and VTA were not significant.

**Table 2:** Statistical values of rest-state functional connectivity comparison in the left substantia nigra (SN).

**Areas**	**MNI coordinates (mm)**			**# Voxels**	**T value**
	**x**	**y**	**z**		
TBI < HC
SMFG	−12	51	18	67	−4.6247
L.AG	−45	−66	36	53	−4.771

*TBI, traumatic brain injury; HC, healthy control; SMFG, superior medial frontal gyrus; L, left; AG, angular gyrus*.

### Substantia Nigra (SN) Volumes

**Figure 2:**
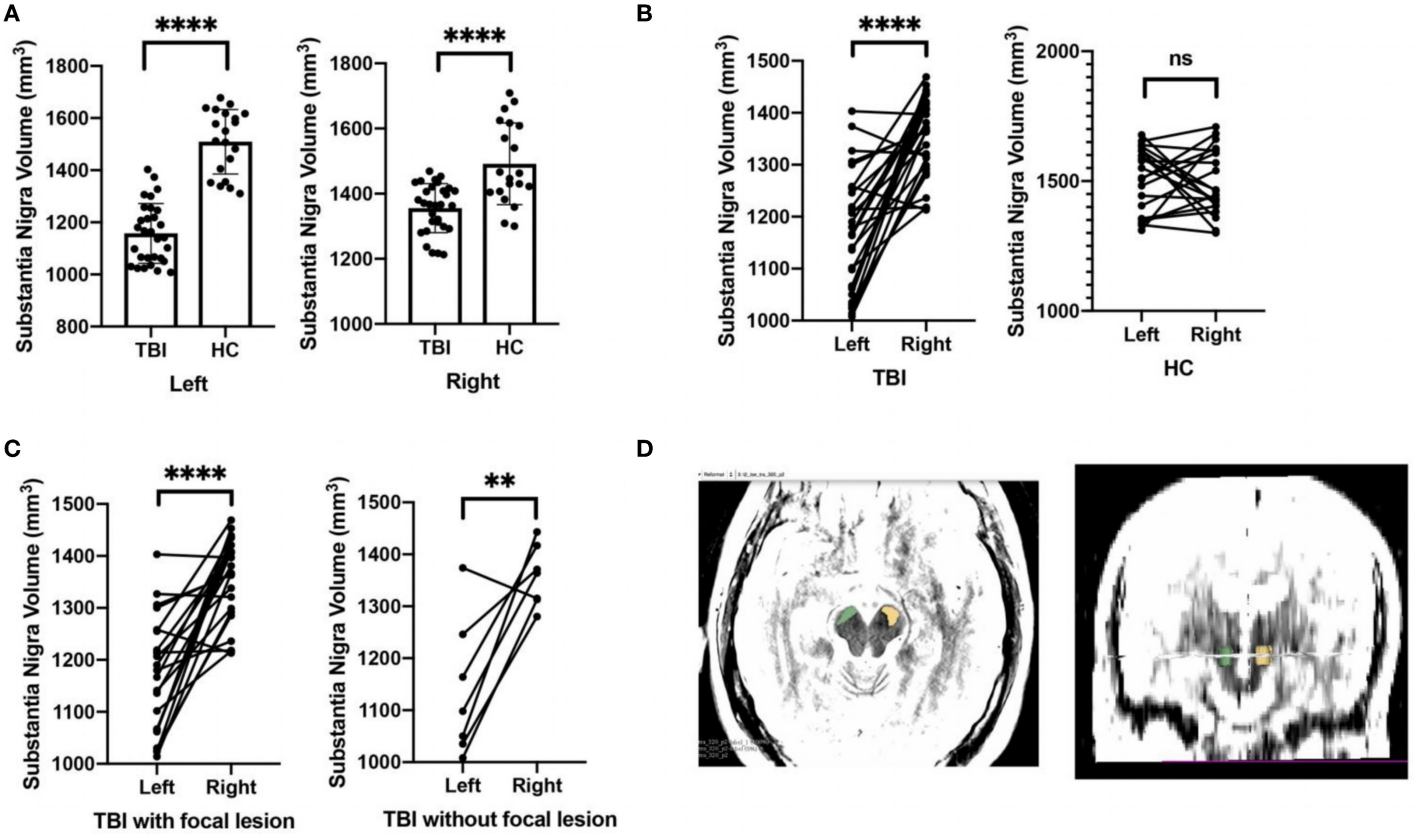
Comparisons on the substantia nigra volume between TBI patients and healthy controls. **(A)** Relative to healthy controls, TBI patients had smaller substantia nigra in both right and left sides. **(B)** For TBI patients, the left substantia nigra was relatively small in contrast with the right one. **(C)** The volume difference between right and left substantia nigra was shown in all types of TBI participants, with and without focal brain injury. **(D)** The example of the hand-drawn substantia nigra on T2-weighted MRI was demonstrated. Green, right substantia nigra; yellow, left substantia nigra. ****p < 0.0001, **p < 0.01.

The patients with TBI had smaller SN in both right and left sides compared to healthy controls. In the TBI group, the left SN was relatively small in contrast with the right one. Moreover, the volume difference between right and left SN was shown in all types of the participants with TBI, with and without focal brain injury (Figure 2).

### Associations of Substantia Nigra (SN) Functional Connectivity With Anxiety and Depressive Symptoms

Patients with TBI are likely to experience the comorbidities of anxiety and depression. Correlation analysis demonstrated a positive association between anxiety and depressive symptoms in the TBI group (*r* = 0.743, *p* = 0.000), in keeping with prior data (27, 28). While exploring the association of anxiety symptoms with functional connectivity, the confounding effect of depressive symptoms cannot be neglected. Similarly, such consideration should be made, investigating the correlation of functional connectivity with depressive symptoms. The functional connectivity between left SN and left angular gyrus was positively associated with post-traumatic anxiety symptoms (β = 0.456, *p* = 0.001), controlling gender, age, education, TIV, and HADS depression. Moreover, the functional connectivity between left SN and left angular gyrus was negatively associated with post-traumatic depressive symptoms (β = −0.323, *p* = 0.047), adjusting for gender, age, education, TIV, and HADS anxiety (Table 3). In healthy controls, no statistical significance was observed.

**Table 3:** Associations of left SN functional connectivity with anxiety and depressive symptoms in patients with TBI and healthy controls.

**Functional connectivity**		**HADS anxiety^a^**				**HADS depression^b^**			
		**TBI**		**Healthy controls**		**TBI**		**Healthy controls**	
		***β***	***p***	***β***	***p***	***β***	***p***	***β***	***p***
Left substantia nigra	SMFG	−0.138	0.329	0.204	0.401	0.129	0.408	−0.245	0.345
Left substantia nigra	L.AG	0.456	0.001	−0.296	0.225	−0.323	0.047	0.266	0.314

a *Multiple linear regression (dependent variable: HADS anxiety; independent variables: functional connectivity, gender, age, education, TIV, and HADS depression)*.

b *Multiple linear regression (dependent variable: HADS depression; independent variables: functional connectivity, gender, age, education, TIV, and HADS anxiety)*.

*TBI, traumatic brain injury; HADS, hospital anxiety and depression scale; SMFG, superior medial frontal gyrus; L, left; AG, angular gyrus*.

## Discussion

In the present study, the patients with TBI reported more anxiety and depressive symptoms. More importantly, some structural and functional alterations, such as smaller SN and reduced functional connectivity in the left SN, were seen in individuals with TBI. Among these findings, the functional connectivity between left SN and left angular gyrus was positively associated with post-traumatic anxiety symptoms and was negatively associated with post-traumatic depressive symptoms.

Experiments in rodents indicate that VTA and SN are both anatomically and functionally distinct (29). Neuroimaging findings further support the idea with the fact that there is a differentiation of connectivity patterns across the VTA and SN in humans (22, 23). Compared to the VTA, the SN demonstrates greater functional coupling with cortical gyri, while the VTA showed greater functional coupling with subcortical structures. In the present study, we identified that the left SN had reduced functional connectivity with medial frontal gyrus, anterior cortical gyri, angular gyrus, and inferior parietal lobule, following TBI. Of note, all these regions with weakened functional connectivity were located in the cortical gyrus. The patients with TBI presented the focal brain lesions mainly in the bilateral orbitofrontal and left temporal cortical regions. On visual inspection, the majority of the areas with altered functional connectivity did not overlap with the lesion regions, indicating that the reduced functional connectivity of left SN was not primarily the result of the lesioned cortex.

Given the occurrence of deforming forces generated by the acceleration and deceleration of the brain during trauma, patients with TBI are apt to suffer from diffuse axonal injury (DAI) (30). The TBI triggers the accumulation of amyloid beta-protein and hyperphosphorylated tau protein (P-tau) in the axon and dendrite, and these aberrant proteins gradually spread to the distal soma, resulting in toxic damage and neuronal loss (31–33). Besides, neuronal loss following TBI may be a result of axonal trauma, which leads to slowly progressive Wallerian degeneration (34). As previously reported (35), individuals with TBI demonstrated reduced volume of the bilateral substantia nigra. What is more, no matter whether they experienced focal brain injuries or not, all the patients with TBI showed smaller SN than healthy controls did. We argued that the altered SN in our patients with TBI might be a consequence of the direct damage to the midbrain nuclei and a consequence of progressive degeneration due to DAI.

The human brain has an overall leftward posterior and rightward anterior asymmetry, which is associated with gender and age (36). Existing research confirms the phenomenon of asymmetry of the brain in the normal aging or neuropsychiatric and neurodegenerative disorders, and the left hemisphere may be predominantly affected in pathological conditions (37). For example, patients with PD show leftward lateralization of SN degeneration (38, 39). Interestingly, the pattern of structural alterations in SN found in our study is similar to that reported in PD. In keeping with the structural asymmetry, leftward lateralization of SN functional connectivity was observed in the patients with TBI as well. To our knowledge, it is the first observation on the lateralization of SN following TBI. For the nature of leftward lateralization of SN degeneration observed in the present study, the control of the dominant hand in right-handers might be a potential explanation, to some extent, which arouses increased neuronal activity that might be detrimental. The exact mechanisms need to be further explored.

Existing data establish links of prefrontal cortex (PFC) dopamine with both anxiety and depression, involving processing emotion and automatic or implicit regulation of emotion. The clinical observations that anxiety and depression are highly prevalent in PD indicate the associations between dopamine and mood disorders (40, 41). In rodents, a reduced dopamine release in medial PFC is observed, which is related to anxiety and depressive behavior (42–45). While our data demonstrated decreased functional connectivity in the superior medial frontal gyrus (SMFG), correlation analyses suggested that the changed functional connectivity was not associated with anxiety or depressive symptoms. As a key brain region involved in reward value, mood, and emotion, the PFC displays considerable anatomical and functional heterogeneity. Accumulating neuroimaging findings suggest that the orbitofrontal and ventromedial frontal cortex plays an important role in the pathology of mood disorders (46, 47). The potential roles of SMFG in the pathology of post-traumatic mood disorders need further verification.

Lying at the intersection of sensory association areas, such as visual, auditory, and visuomotor areas, the angular gyrus is an important commissural area in the posterior part of the brain and belongs to the frontoparietal control network (FCN) (48). The FCN is thought to be a functional hub both generally and specifically in terms of distributed connectivity to many diverse brain networks, such as default mode networks, dorsal attention networks, and salience networks (49). Recent pieces of research have supported the notion that the abnormal FCN may be a common feature across many diseases, including schizophrenia, depression, and anxiety (50, 51). A decreased connectivity within the FCN and disturbed connectivity between the FCN and networks involved in internal or external attention may suggest depressive biases toward internal thoughts at the expense of external attentions. Meanwhile, altered connectivity between cognitive control systems and those involved in salience or emotion processing may underlie mood deficits. It is not surprising to observe hypoconnectivity between substantia nigra and angular gyrus among populations with TBI, which is correlated with post-traumatic anxiety and depressive symptoms. Our findings suggest a large-scale network dysfunction might account for the anxiety and depressive symptoms following TBI.

We analyzed the limitations of our research also for further improvements. First, the population enrolled in this study was relatively small. With the different brain-injured regions of patients, the degree of injury is not consistent, which may lead to high heterogeneity among patients and impact on the results. Second, ROI is modeled as a sphere by a coordinate method according to the template, which inevitably deviates from the structure and location of the actual brain area and influences the functional connectivity calculation. Moreover, dopaminergic nerve fibers are not the only nerve fibers projected from the VTA and SN. We cannot distinguish different types of nerve fibers based only on the MRI image or data. Finally, the HADS scale is a self-reported questionnaire. Patients with TBI with cognitive impairment may have a tendency to overestimate or underestimate their mood problems.

## Conclusion

The patients with TBI presented smaller SN and reduced functional connectivity in the left SN relative to healthy controls. Functional connectivity between left SN and left angular gyrus was positively associated with post-traumatic anxiety symptoms and negatively associated with post-traumatic depressive symptoms. Impaired mesocortical functional connectivity might be implicated in post-traumatic anxiety and depression.

## Data Availability Statement

The raw data supporting the conclusions of this article will be made available by the authors, without undue reservation.

## Ethics Statement

The studies involving human participants were reviewed and approved by the Ethics Committee of the Pudong New Area People's Hospital. The patients/participants provided their written informed consent to participate in this study.

## Author Contributions

YD, QX, SG, and JH drafted the submitted work. All the authors revised it critically for important intellectual content and provided approval for publication of the content. All authors contributed to the article and approved the submitted version.

## Funding

This work was supported by the National Natural Science Foundation of China (Grants 81671227, 81671201).

## Conflict of Interest

The authors declare that the research was conducted in the absence of any commercial or financial relationships that could be construed as a potential conflict of interest.

## Publisher's Note

All claims expressed in this article are solely those of the authors and do not necessarily represent those of their affiliated organizations, or those of the publisher, the editors and the reviewers. Any product that may be evaluated in this article, or claim that may be made by its manufacturer, is not guaranteed or endorsed by the publisher.
